# Fluoroquinolone-resistant *Escherichia coli* Carriage in Long-Term Care Facility

**DOI:** 10.3201/eid1106.041335

**Published:** 2005-06

**Authors:** Joel N. Maslow, Betsy Lee, Ebbing Lautenbach

**Affiliations:** * Veterans Affairs Medical Center, Philadelphia, Pennsylvania, USA;; † University of Pennsylvania, Philadelphia, Pennsylvania, USA

**Keywords:** molecular epidemiology, E.coli, drug resistance, bacterial, fluoroquinolone, nursing home, long term care

## Abstract

We conducted a cross-sectional study to determine the prevalence of, and risk factors for, colonization with fluoroquinolone (FQ)-resistant *Escherichia coli* in residents in a long-term care facility. FQ-resistant *E. coli* were identified from rectal swabs for 25 (51%) of 49 participants at study entry. On multivariable analyses, prior FQ use was the only independent risk factor for FQ-resistant *E. coli* carriage and was consistent for FQ exposures in the previous 3, 6, 9, or 12 months. Pulsed-field gel electrophoresis of FQ-resistant *E. coli* identified clonal spread of 1 strain among 16 residents. Loss (6 residents) or acquisition (7 residents) of FQ-resistant *E. coli* was documented and was associated with de novo colonization with genetically distinct strains. Unlike the case in the hospital setting, FQ-resistant *E. coli* carriage in long-term care facilities is associated with clonal spread.

The increasing prevalence of antimicrobial resistance affecting hospitalized and ambulatory populations has gained national prominence. Although this setting is less well studied, evidence is mounting that antimicrobial resistance is also an increasing problem in long-term care facilities (1–5). Most research on colonization with resistant bacteria in the long-term care setting has focused on gram-positive organisms, in particular, methicillin-resistant *Staphylococcus aureus* and vancomycin-resistant *Enterococcus faecalis* and *E. faecium* (6–9); substantially fewer data address the prevalence of antimicrobial resistance among gram-negative bacteria.

Past studies in such facilities found that resistance in gram-negative bacteria was not uncommon, whereas resistance among isolates of *Escherichia coli* was unusual (1,4,10,11). More recent investigations reported that among hospitalized patients, residence in a long-term care facility was a risk factor for colonization or infection with *E. coli* that was resistant to higher generation cephalosporins and to the fluoroquinolone (FQ) antimicrobial agents (12–15). Moreover, Weiner et al. reported that nursing home residents were likely to be colonized with such isolates at the time of hospital admission (15). Finally, we recently noted significant increases over a 5-year period in FQ-resistant *E. coli* in clinical isolates from 4 long-term care facilities in Pennsylvania (16). *E. coli* is the most common species causing infections in the elderly long-term care resident, primarily as a consequence of the prevalence of urinary tract infections. FQs are the most frequently prescribed antimicrobial class in this setting, accounting for ≈25% of all antimicrobial prescriptions (17,18).

While evidence suggests that the prevalence of FQ-resistant *E. coli* carriage among such residents is increasing, no patient level study of risk factors for FQ-resistant *E. coli* colonization has been performed in this setting (16). We conducted this study to determine the prevalence of fecal carriage with FQ-resistant *E. coli* among residents of a single long-term care facility, to identify risk factors associated with colonization, and to describe the ecology of carriage of FQ-resistant *E. coli* over time.

## Methods

### Study Site and Patient Population

This study was conducted at a single Veterans Affairs Medical Center nursing home. This 240-bed facility, adjacent to a 150-bed acute-care hospital, opened in 1990 (9) and maintains an average daily census of >95% of capacity. The demographics of the facility parallel that of the adjacent tertiary medical center: 1% female and 50% minority residents. More than 80% of residents are admitted from the adjacent medical center. Approximately 20 beds are used for patients requiring admission for skilled nursing care.

Residents were recruited for this study from March to July 2002 (19). All residents were considered eligible for inclusion if informed consent was obtained. For residents who were cognitively impaired, informed consent was obtained from a legal guardian or medical proxy. The study was reviewed and approved by the local institutional review board.

For enrolled participants, rectal swabs were obtained at study entry. FQ-resistant *E. coli* were detected by a 1-step screening procedure (19). Species identification and FQ resistance were confirmed by automated testing (Vitek, bioMérieux, Hazelwood, MO, USA). Because a recent study noted excellent sensitivity and specificity (>90%) for rectal swab specimens compared to stool culture for detecting FQ-resistant *E. coli* (20), subsequent rectal swab samples were obtained at monthly intervals to identify changes in colonization status.

Case-patients were defined as those for whom FQ-resistant *E. coli* was identified at the initial sampling. Controls were defined as patients without FQ-resistant *E. coli* at the initial sampling. Any study participant colonized with both a FQ-resistant *E. coli* and a FQ-susceptible *E. coli* was considered a case-patient. Patients with new colonization with FQ-resistant *E. coli* were defined as those for whom the initial study sample yielded only FQ-susceptible *E. coli* with a sample at a later time point yielding FQ-resistant *E. coli*. Patients clearing colonization with FQ-resistant *E. coli* were defined as those for whom this organism was detected at the time of initial sampling with 2 subsequent consecutive samples that yielded only FQ-resistant *E. coli*.

### Data Collection

Computerized medical records were reviewed for all patients. Patients admitted to the long-term care facility are evaluated by a nurse practitioner and physician with a comprehensive assessment documented at admission and at yearly intervals. Quarterly assessments are performed for minimal data set, functional, and mental evaluations. Interval notes by the nurse practitioner and physician were entered at times of clinical events. Data collection was assisted by the fact that patients requiring hospital admission were cared for in the adjacent medical center. Medical records for both facilities are maintained jointly. The nursing home admission note and yearly review notes contained detailed problem lists. For patients who had received care at other Veterans Affairs institutions, medical records were available through the Veterans Affairs Intranet. Demographic data obtained included age, sex, race, date of admission to the facility, and dates of prior hospitalizations at the time of study enrollment.

Records were also reviewed to identify potential risk factors for carriage of FQ-resistant *E. coli*. Devices and conditions that would interfere with normal mucosal defense mechanisms (21) were ascertained, including indwelling catheters, intravenous catheters, feeding tubes, decubitus ulcers, and surgical wounds. Data on coexisting conditions included renal insufficiency (defined as a serum creatinine level of >2.0), liver failure, hepatitis C, cirrhosis, congestive heart failure, chronic obstructive lung disease, malignancy, and HIV. Disorders associated with cognitive impairment included dementia, history of cerebral vascular accident, and psychiatric disorders such as depression and schizophrenia. Low ambulatory status was defined as requiring a wheelchair for ambulation or documentation of the patient's being bed-bound. Pharmacy records were reviewed for all antimicrobial use in the year before study entry and during the period of prospective fecal sample collection.

### Genotypic Analysis of *E. coli*

Up to 25 colonies of *E. coli* as available were sampled from the initial patient sample, and ≤10 colonies were obtained from subsequent cultures. Individual colonies were subjected to pulsed-field gel electrophoresis (PFGE) to determine macrorestriction polymorphisms after *Xba*I restriction digestion of chromosomal DNA as described (22,23). Clonal analysis was performed (24) per the criteria of Tenover et al. (25); isolates that differed by ≤3 bands were considered clonal and isolates that differed by 4 to 6 bands were considered related.

### Statistical Methods

We conducted a cross-sectional study to identify risk factors for FQ-resistant *E. coli* colonization. Bivariable analysis was conducted to determine the association between potential risk factors and such colonization; the primary risk factor variable of interest was prior FQ use. Although we used FQ use in the past year as the primary measure, we also explored different cutpoints of prior FQ use (i.e., 3, 6, and 9 months). Categorical variables were compared by using the Fisher exact test. An odds ratio (OR) and 95% confidence interval (CI) were calculated to evaluate the strength of associations. Continuous variables were compared with the Student *t* test or the Wilcoxon rank-sum test, depending on the validity of the normality assumption (26).

Stratified analyses were then performed to identify where confounding and interaction were likely to exist for the primary comparison of interest (i.e., FQ exposure and FQ-resistant *E. coli* colonization). Stratification was performed with the following variables: duration of residence in the facility before study enrollment (divided into quartiles) and hospitalization in the prior year. The Mantel-Haenszel test for summary statistics was used to evaluate possible confounding (27); interaction was assumed when the test for heterogeneity between the OR for different strata was significant (p<0.05).

Multivariable analysis was performed with multiple logistic regression (28). Building the multivariable model began with inclusion of key variables based on a priori hypotheses (i.e., prior FQ use). All variables with a p value <0.20 on bivariable analysis were also considered for inclusion in a multivariable explanatory model (29). Variables were also considered for inclusion in the model if they were noted to be involved in confounding on stratified analysis. Finally, we evaluated the impact of the variable indicating "time at risk" (i.e., duration of residence in the facility before study enrollment) in the multivariable model. The interaction between risk factor variables in the final model was also investigated. A 2-tailed p value <0.05 was considered significant. All statistical calculations were performed with standard programs in STATA v. 8.0 (Stata Corp, College Station, TX, USA).

## Results

Of 75 randomly selected residents who were consecutively approached for enrollment, 60 (80%) gave informed consent for inclusion in the study. Five residents were discharged or died before having an initial rectal sample obtained for study; 6 other residents had stool samples that did not yield *E. coli* despite multiple samplings. Thus, samples from 49 residents yielded *E. coli* isolates and were included in the study. The median age of participants was 69 years (range 38–98 years). Two (4.1%) participants were female, 18 (36.7%) were African American, and 1 (2.0%) was Hispanic. Three residents were admitted only for skilled nursing care. Patient functional scores exhibited a bimodal pattern: one third of patients were considered full care, one third as fully independent, and the remaining patients evenly spread through a range of functional levels. Approximately 50% of patients were incontinent. Multiple sclerosis was documented for 5 patients.

FQ-resistant *E. coli* was detected in the stool of 25 (51%) residents (19). The median age of case-patients was 73 years (range 38–87 years) and 65.5 years (range 42–98 years) for controls (p = 0.99). One case-patient and 1 control were women (p>0.99). Ten case-patients and 8 controls were African American (p = 0.77). The results of bivariable analysis are shown in Table 1. Duration of nursing home residence, hospitalization within the 12 months before study entry, low ambulatory status, FQ use within the past year, and prior metronidazole use were associated with FQ-resistant *E. coli* colonization. Association of FQ exposure and colonization with FQ-resistant *E. coli* was noted at all quartiles except for exposure within the 3 months before study entry: 6 (25%) of 24 controls and 18 (72%) of 25 case-patients received FQ in the 9 months before study entry (p = 0.002); 5 (21%) of 24 controls and 14 (56%) of 25 case-patients received FQ in the 6 months before study entry (p = 0.02); and 3 (13%) of 24 controls and 7 (28%) 25 received FQ in the 3 months before study entry (p = 0.29).

**Table 1:** Comparison of cases and controls, fluoroquinolone-resistant *Escherichia coli* colonization study, long-term care facility*

Variable	Controls (n = 24) (%)	Cases (n = 25) (%)	OR (95% CI)	p value†
Prior hospitalization	18/24 (75.0)	13/25 (52.0)	0.36 (0.09, 1.41)	0.08
Duration of residence in facility (d)‡§	209.5 (22–2,571)	411 (81–2,580)		0.13
Decubitus ulcer	6/24 (25.0)	2/25 (8.0)	0.26 (0.02, 1.73)	0.14
Low ambulatory status	15/24 (62.5)	10/25 (40.0)	0.40 (0.11, 1.46)	0.16
Fluoroquinolone use	6/24 (25.0)	18/25 (72.0)	7.71 (1.86, 33.60)	0.002
Metronidazole use	1/24 (4.2)	7/25 (28.0)	8.90 (0.96, 420.17)	0.05

*OR, odds ratio; 95% CI, 95% confidence interval. †Fisher exact test (categorical variables); Wilcoxon-rank sum test (continuous variables). ‡Median (range). §Days from admission into facility until study enrollment.

On multivariable analysis, only prior FQ use remained an independent risk factor for FQ-resistant *E. coli* colonization (Table 2). A borderline significant association was seen between FQ-resistant *E. coli* colonization and duration of prior long-term care residence as well as prior metronidazole use.

**Table 2:** Multivariable comparison of cases and controls, fluoroquinolone-resistant *Escherichia coli* colonization, long-term care facility study*

Variable	Unadjusted OR	Adjusted OR (95%CI)	p value
Fluoroquinolone use	7.71	9.16 (2.08, 40.41)	0.003
Duration of residence in facility†§		1.00 (1.00, 1.01)	0.07
Metronidazole use	8.90	5.90 (0.52, 66.50)	0.15

*CI, confidence interval. †Days from admission into facility until study enrollment. §Odds ratio (OR) reflects the odds associated with each increase in 1 day of residence in the facility.

Genotypic analysis of 25 colonies from each study participant was performed by PFGE. Multiple strains were detected in initial fecal samples from 22 (44.9%) participants. For those with FQ-resistant *E. coli* in stool, 16 (64%) had multiple *E. coli* strains. In contrast, multiple strains were less common for those not colonized with FQ-resistant *E. coli* as only 6 (25%) harbored multiple strains of *E. coli* (OR 5.33, 95% CI 1.34–22.23, p = 0.006). Both FQ-resistant and FQ-susceptible *E. coli* were detected in fecal samples from 15 participants. Comparison of strains between participants documented 2 clusters of clonally related strains of FQ-resistant *E. coli* detected for multiple persons. Clone A was detected in fecal samples from 16 participants: 7 were colonized with clone A alone and 9 with strain A and ≥1 unique strains of FQ-resistant *E. coli*, FQ-susceptible *E. coli*, or both (Table 3). A second resistant clone, clone C, was detected in the stools from 2 participants (Table 3). Unique stains of FQ-resistant *E. coli* (i.e., other than clone A or C) were detected in the stools of 14 (56%) persons. FQ-susceptible strains were genetically unique in different participants. FQ exposure in patients colonized with clone A compared with other strains of FQ-resistant *E. coli* did not differ (data not shown). Thus, person-to-person clonal spread of FQ-resistant *E. coli* was common and occurred in the absence of FQ exposure.

**Table 3:** Genotypic analysis of patient samples of fluoroquinolone-resistant Escherichia coli*

Analysis	No. of patients
Strain A detected	15
Strain A only detected	7
Strain A + FQSEC	3
Strain A + FQSEC + unique FQREC	5
Strain B detected	2
Strain B + FQSEC	1
Strain B + FQSEC + unique FQREC	1
Unique FQREC detected	7
Unique FQREC only	2
Unique FQREC + FQSEC	5

*FQSEC, fluoroquinolone-susceptible *E. coli*; FQREC, fluoroquinolone-resistant *E. coli*.

Of the 49 participants enrolled in the study, 45 (92%) had follow-up cultures. For the 25 participants initially colonized with FQ-resistant *E. coli*, 22 (88%) had sequential follow-up cultures (median follow-up 6 months, range 1–10 months). Rectal swabs from 16 (73%) study participants continued to yield FQ-resistant *E. coli* at each monthly sample, while swabs from 6 patients demonstrated clearance of this organism. The median time for clearance of FQ-resistant *E. coli* was 5 months (range 2–10 months). For the 24 participants not initially colonized with FQ-resistant *E. coli*, 23 (96%) had follow-up rectal swab samples. New colonization with FQ-resistant *E. coli* was detected in samples from 7 (30%) persons at a median of 6 months from study entry (range 1–8 months). Three of the 45 participants included in this follow-up phase were prescribed antimicrobial agents after study entry, but the antimicrobial agent was not an FQ. For these 3 patients, no change in carriage of FQ-resistant *E. coli* occurred. No study participant was hospitalized in the follow-up period. No demographic or clinical factors were associated with a change in colonization status. Thus, resistance patterns were altered in a large number of study participants (13 [29%] of 45), independent of antimicrobial treatment in the 1-year follow-up period.

For the 7 study participants with newly acquired FQ-resistant *E. coli*, PFGE genotypes of all strains of FQ-susceptible *E. coli* cultured from the initial study sample were compared to 5 colonies of FQ-resistant *E. coli* randomly chosen from the first sample yielding FQ resistance. For each patient, PFGE genotypes differed between initial and subsequent samples, a demonstration of de novo colonization with resistant bacteria. Similarly, clearance of FQ-resistant *E. coli* was associated with de novo colonization with genetically distinct strains in 5 of 6 cases. For 1 case-patient, a resistant strain cleared; colonization with a susceptible strain present at the initial study visit continued.

## Discussion

FQ use was the only independent risk factor for FQ-resistant *E. coli* colonization. Borderline significant associations existed between carriage of such organisms and duration of residence in the long-term care facility before study enrollment and prior metronidazole use. Most study participants harboring FQ-resistant *E. coli* were colonized with clonally related strains. Change in colonization status, either acquisition or clearance of FQ-resistant *E. coli*, was common in the 1-year period of follow-up and did not appear to be related to antimicrobial therapy.

Although never previously investigated in a long-term care facility, the association between FQ exposure and colonization or infection with FQ-resistant *E. coli* has been documented (30–36). Other investigators have, however, found prior FQ exposure to represent a modest (37) or no risk (38) for colonization with FQ-resistant *E. coli*. Exposure effect was found to be relatively short-lived among cancer patients prescribed FQ antimicrobial agents as part of prophylaxis during chemotherapy: >75% of patients had clearance of FQ-resistant *E. coli* within 3 months of ceasing FQ use (31,34). While our data corroborate the relationship between FQ exposure and FQ-resistant *E. coli*, we also found that temporally more distant FQ exposures (>3 months) may also represent risks for colonization with resistant bacteria. And, in contrast to the findings with cancer patients, colonization with FQ-resistant *E. coli* may persist over long periods.

We addressed the question of clearance and acquisition of FQ-resistant *E. coli* in the long-term care setting. Of the 45 patients with follow-up cultures, 13 (29%) demonstrated acquisition or clearance with FQ-resistant *E. coli* over the 1-year follow-up period. No patient who changed colonization status was treated with FQ during the 1-year study period or in the year before study entry (data not shown). Thus, in this closed setting, colonization with FQ-resistant *E. coli* appears to be a dynamic process and may be less affected by prior FQ therapy than it would be in the acute-care setting.

Molecular analysis shed further light on this process. In all cases but one, alteration in colonization status (whether from resistant to susceptible or susceptible to resistant) was marked by de novo colonization with bacteria genetically distinct from those patients had at study entry. For the remaining patient, a resistant strain was cleared, and a susceptible strain detected at study entry persisted.

Most (64%) study participants colonized with FQ-resistant *E. coli* harbored a single clonally related strain (clone A); 56% were colonized with strains other than clone A. Thus, the emergence of FQ-resistant *E. coli* colonization in this patient population appeared to arise from patient-to-patient spread as well as de novo resistance. Clonal spread of FQ-resistant *E. coli* has not been adequately addressed, although 2 studies of cancer patients suggest that it is uncommon in other clinical settings (34,39).

Our study had several potential limitations. Our small sample size may have hampered our ability to identify smaller effect sizes for risk factors of interest. The possibility of selection bias is of concern, given that only 25% of the total population of the facility was enrolled in the study. Since we only sampled participants monthly, the longitudinal component of the study is limited regarding fully characterizing the dynamics of how frequently resistance profiles of nursing home residents are altered. These factors also limit our ability to assess outcomes and risks for subsequent infections. Changes in resistance profiles were also associated with colonization with different *E. coli* strains. Whether this represents possible antimicrobial effects on non–*E. coli* affecting the ability of new strains of *E. coli* to colonize the gut is unknown. Since environmental cultures were not performed, we cannot exclude a common source exposure (e.g., food or showers) to explain a single clone's being detected among different patients. Finally, whether our study results can be generalized to other institutions is not known.

Our study represents the first investigation of patient-level risk factors for FQ-resistant *E. coli* colonization in the long-term care setting. We found that FQ-resistant *E. coli* carriage is common in such residents and that prior FQ exposure is the only independent risk factor for such carriage. These finding emphasize the importance of limiting antimicrobial drug use in general and FQ use in particular in this setting. Unlike the hospital setting, carriage of FQ-resistant *E. coli* in long-term care facilities is associated with clonal spread. Finally, carriage of FQ-resistant *E. coli* in long-term care facilities appears to represent a fluid process, with frequent loss or acquisition of FQ-resistant *E. coli*.
